# Investigation of Separating Temperature-Induced Structural Strain Using Improved Blind Source Separation (BSS) Technique

**DOI:** 10.3390/s24248015

**Published:** 2024-12-15

**Authors:** Hao’an Gu, Xin Zhang, Dragoslav Sumarac, Jiayi Peng, László Dunai, Yufeng Zhang

**Affiliations:** 1 Department of Engineering Mechanics, Hohai University, Nanjing 210098, China; mechgha@hhu.edu.cn; 2 Department of Measurement and Control, Nanjing University of Posts and Telecommunications, Nanjing 210003, China; xzhang@njupt.edu.cn; 3 School of Civil and Surveying & Mapping Engineering, Jiangxi University of Science and Technology, Ganzhou 341000, China; dragosumi@gmail.com; 4 Faculty of Civil Engineering, University of Belgrade, 11000 Belgrade, Serbia; 5 The State Key Laboratory for the Safety, Long-Life, Health Operation and Maintenance of Long-Span Bridges, Jiangsu Provincial Institute of Traffic Science (JSTI Group), Nanjing 210098, China; pjiayi@126.com; 6 Department of Structural Engineering, Faculty of Civil Engineering, Budapest University of Technology and Economics, 1111 Budapest, Hungary; dunai.laszlo@emk.bme.hu

**Keywords:** structural health monitoring, bridge strain separation, temperature effect, temperature-induced strain, blind source separation, second-order blind identification, strain gauges

## Abstract

The strain data acquired from structural health monitoring (SHM) systems of large-span bridges are often contaminated by a mixture of temperature-induced and vehicle-induced strain components, thereby complicating the assessment of bridge health. Existing approaches for isolating temperature-induced strains predominantly rely on statistical temperature–strain models, which can be significantly influenced by arbitrarily chosen parameters, thereby undermining the accuracy of the results. Additionally, signal processing techniques, including empirical mode decomposition (EMD) and others, frequently yield unstable outcomes when confronted with nonlinear strain signals. In response to these challenges, this study proposes a novel temperature-induced strain separation technique based on improved blind source separation (BSS), termed the Temperature-Separate Second-Order Blind Identification (TS-SOBI) method. Numerical verification using a finite element (FE) bridge model that considers both temperature loads and vehicle loads confirms the effectiveness of TS-SOBI in accurately separating temperature-induced strain components. Furthermore, real strain data from the SHM system of a long-span bridge are utilized to validate the application of TS-SOBI in practical engineering scenarios. By evaluating the remaining strain components after applying the TS-SOBI method, a clearer understanding of changes in the bridge’s loading conditions is achieved. The investigation of TS-SOBI introduces a novel perspective for mitigating temperature effects in SHM applications for bridges.

## 1. Introduction

With the continuous advancements in bridge construction techniques, the number of large-span steel box girder cable-stayed bridges has witnessed a significant increase. These structures offer numerous advantages, including enhanced economic efficiency and environmental sustainability, while maintaining robust load-bearing capabilities—a crucial aspect to accommodate the growing demands for transportation infrastructure. However, these structures also face challenges due to cyclic loading from heavy vehicular traffic, compounded by environmental factors such as temperature variations and wind. In the complex environments of river and coastal areas, large-span steel box girder cable-stayed bridges are subjected to alternating stresses and electrochemical reactions. Consequently, critical stress zones become susceptible to fatigue cracking and stress corrosion, which can potentially lead to catastrophic failures [1,2]. Given the inherent maintenance difficulties associated with large-span bridges, many of these structures are equipped with structural health monitoring (SHM) systems that provide timely assessments of bridge safety. Among the various parameters monitored, the identifications of load and strain conditions are particularly vital, as these factors directly reflect the bridge’s current performance. These feedbacks are essential not only for understanding changes in load-carrying capacity but also for indirectly indicating structural damage. Considering that structural damage identification is a research focus of growing interest [3], the analysis and processing of strain data offer significant value in the context of bridge management.

Currently, bridge strain monitoring methods include strain gauges, digital imaging [4,5], microwave interference radar [6,7], and other techniques [8]. Among these methods, strain gauges are considered the most economical and efficient approach for recognizing bridge strain, while also maintaining a high level of accuracy. This makes them the predominant method in use today [9]. Meanwhile, the technology for strain monitoring utilizing strain gauges is progressively advancing, marked by the emergence of novel gauge types crafted from innovative materials [10,11], along with advancements that seamlessly integrate big data technology [12]. However, strain gauges are susceptible to temperature effects, which refer to the phenomenon where temperature changes cause variations in the resistance of strain gauges, as

(1) $$(\frac{∆R}{R})=\alpha_{t}∆t+K(\beta_{s}\;-\beta_{t})∆t,$$

where $\alpha_{t}$ represents the temperature coefficient of resistance of strain gauge materials, K denotes the sensitivity coefficient of the strain gauge, while $\beta_{s}$ and $\beta_{t}$ are the linear expansion coefficient of the test piece and the strain gauge, respectively.

As a result, the strain measurements obtained using strain gauges may be influenced by ambient temperature fluctuations, which can result in inaccuracies when representing the actual loading conditions of the bridge. Additionally, temperature variations have the potential to alter the material properties of the bridge to a certain degree [13,14], thereby potentially obscuring crucial information regarding the bridge’s current performance. By disregarding other non-primary components of strain, such as shrinkage strain and creep strain, the strain measured by strain gauges can be formulated by the following equation as [15]

(2) $$\varepsilon \left(t\right)=\varepsilon_{T}\left(t\right)+{\;\varepsilon }_{V}\left(t\right),$$

where ε(t), $\varepsilon_{T}\left(t\right)$, and $\varepsilon_{V}\left(t\right)$ represent the measured structural strain, the temperature-induced strain, and the vehicle-induced strain, respectively. Indeed, eliminating temperature effects from the measured strain data is a crucial aspect of bridge health monitoring.

Thus far, scholars have proposed diverse solutions for separating temperature effects from the total strains in bridges, which can be broadly categorized into two approaches: mapping model separation and direct algorithm separation [16]. The first category focuses on the development of statistical models that accurately capture the relationship between temperature and structural strain. By utilizing measured temperature data to calculate the temperature-induced strain response, this method enables the removal of temperature effects from the strain measurements. Several scholars have derived formulas to establish the relationship between temperature distribution and temperature-induced strain in bridges [17,18]. Sun [19] introduced a temperature–strain regression model that incorporates three types of temperature data collected from an actual bridge SHM system. Subsequently, a time-varying average method was utilized to effectively minimize the temperature-induced strain effects from the strain data. Similarly, Kromanis [20] presented a regression-based methodology to capture the relationships between temperature distributions and structural responses, using a generic computational framework that predicted the thermal response of structures using distributed temperature measurements. Wang [21] developed an enhanced Bayesian dynamic linear model, grounded in a temperature-driven baseline, to analyze and forecast the relationship between temperature and temperature-induced strain. Various scholars have conducted studies related to the calculation of temperature fields in bridges [22,23]. In recent years, neural network models have been employed to develop temperature-induced strain models, demonstrating significant effectiveness in investigating the temperature–strain relationship due to their superior adaptive learning capabilities and ability to process large datasets. Fang [16] proposed a temperature–strain mapping modeling method for cable-stayed bridges based on transfer learning and bi-directional long- and short-term memory (Bi-LSTM) neural networks. After constructing a Bi-LSTM neural network benchmark model based on the outcome of analytical mode decomposition (AMD) on the measured strain data, transfer learning was utilized to facilitate the formation of the temperature–strain model for the entire bridge. Zhu [24] utilized an adaptive neural network fuzzy inference system to explore the complex nonlinear relationship between measured temperature and strain data. However, the approach of mitigating temperature effects through temperature–strain numerical modeling is often impeded by manual intervention, such as the artificial definition of parameters. Furthermore, these models typically require extensive temperature–strain data specific to the target study bridges, which complicates their generalization to other structures and may compromises the accuracy of the model.

The second category of temperature effect separation entails the direct extraction of temperature-induced strain from measured strain through the application of signal decomposition algorithms [25]. Methods belonging to this category are more straightforward and efficient compared with those in the first category, rendering them more suitable for fulfilling the real-time demands of SHM applications. Wu [26] introduced a multi-resolution stepwise approach for classifying raw strain data in the frequency domain, utilizing the energy spectral ratio index. This methodology effectively minimized temperature-induced strain and isolated the strain information attributed to structural stress. Li [27] employed AMD to differentiate temperature strain based on a cut-off frequency, which was determined by comparing the power spectral densities of temperature strain gauge data and work strain gauge data, between the temperature strain and the dynamic load strain. Tenelema [28] proposed an approach utilizing Principal Component Analysis (PCA) to distinguish structural damage conditions from those alterations arising due to environmental effects in a numerical benchmark bridge. Empirical mode decomposition (EMD) and its derivatives are frequently utilized in relevant studies. These techniques decompose dynamic strains into intrinsic mode function (IMF) sequences for temperature effect separation after a specific process [29]. Chen [30] treated temperature-induced strains as a trend component within the original strain data and subsequently used the EMD method to isolate this temperature trend by segmenting the entire signal into several subsections. This method facilitated more precise calculations of failure probabilities and improved reliability assessments. Xia [15] separated temperature-induced strain from the measured strain responses of a long-span suspension bridge using ensemble empirical mode decomposition (EEMD) technology, thereby enhancing the efficacy of temperature effect separation. Furthermore, Li [29] proposed an advanced and refined EMD method, termed Auto-EMD, to automatically decouple temperature- and vehicle-induced strains from extensive strain data collected in real time. In addition, Saha [31] introduced a multi-parameter sensing system augmented with machine learning (ML) for the concurrent detection of strain and temperature effects, subsequently employing ML algorithms to discern temperature influences from strain variations. Weil [32] developed a filtering method that separates slower, temperature-induced strain variations into filtered data, while categorizing load-induced strain as process noise. However, existing methods in the second category, particularly those based on EMD and its variants, confront challenges associated with subjectivity. The arbitrary selection of thresholds and truncation parameters can considerably influence the accuracy of signal separation. Moreover, in practical engineering scenarios involving vast quantities of monitoring data, these methods often rely on local feature extraction, making them susceptible to inconsistencies and unreliable results, particularly when handling nonlinear and unstable signals [33,34].

The aforementioned problem can be framed as the separation of the temperature-induced signal component from the vehicle-induced signal component within a strain signal that comprises intricate elements. This scenario closely resonates with the principles of blind source separation (BSS), which has exhibited substantial application value in tackling similar challenges across various domains. Lekshmylal [35] employed several novel BSS algorithms to minimize artifacts in electroencephalogram (EEG) signals arising from eye movements and muscle movements and compared their efficacy. Sharma [36] utilized the frequency domain BSS technique to isolate original speech signals from a user’s voice, successfully retrieving the original sound by filtering out external noises and selecting the optimal frequency signal. Li [37] proposed an underdetermined BSS algorithm grounded in density peak clustering for fault diagnosis in mechanical rotating components, addressing the noise sensitivity issue inherent in traditional BSS methods when estimating the number of vibration sources in mechanical systems. Saeid [38] designed a BSS model to recover geochemical signal sources, which was subsequently used to identify multi-depth ore-related enrichment patterns in complex metalorganic systems. Consequently, leveraging BSS techniques to address the challenge of isolating the effects of temperature on bridges represents a notable research direction that has received limited attention in the field of SHM.

Among the diverse types of BSS algorithms, Second-Order Blind Identification (SOBI) [39] stands out as one of the most extensively utilized. It leverages the second-order statistics of the source signals to achieve decorrelation, thereby offering the advantage of not necessitating statistical independence. Moreover, SOBI exhibits greater robustness against signal non-Gaussianity, particularly in noisy environments, rendering it highly suitable for modal analysis and related applications. In recent times, SOBI has found applications in signal unmixing problems spanning multiple disciplines. Oliveira [40] tackled the challenge of distortion interference analysis in voltage signals of distribution networks by utilizing SOBI to decompose voltage and/or current signals into their harmonic and interharmonic component waveforms. This methodology facilitated a clearer evaluation of signal quality and the identification of interference sources within the network. Miao [41] introduced a SOBI-based noise reduction approach for mechanical fault features and successfully implemented it in faulty rotor machinery. In structural engineering, Laventure [42] estimated the aerodynamic damping ratios of concrete chimneys under various windy conditions, employing SOBI to mitigate the interference from wind effects across different scenarios. Consequently, introducing SOBI into the SHM domain holds considerable promise and value.

Hence, to address the aforementioned issue, this paper investigates a novel method termed Temperature-Separate Second-Order Blind Identification (TS-SOBI), which is an advanced and applied version of the SOBI algorithm. The primary objective of TS-SOBI is to separate temperature-induced strains from the original structural strain data gathered through SHM systems. The specific innovations of this paper encompass: (i) a substantial enhancement of the SOBI algorithm, specifically tailored to exclude temperature-induced strains from the bridge loads being analyzed; and (ii) a comprehensive demonstration of the TS-SOBI method, with its feasibility validated through numerical simulations using finite element (FE) modeling and the analysis of real data collected from actual bridge structures. These contributions represent a pioneering approach to integrating robust BSS techniques into data analysis for SHM applications. The ultimate goal is to overcome the limitations associated with traditional methods of mitigating temperature effects, as previously outlined.

The remainder of this paper is organized as follows: Section 2 offers an overview of the fundamental theory supporting the TS-SOBI method, along with a detailed description of its implementation process. Section 3 presents the numerical validation of the TS-SOBI method utilizing an FE bridge model. Section 4 reports on the application of TS-SOBI to actual SHM data from the Sutong Yangtze River Highway Bridge. Lastly, Section 5 presents concluding remarks.

## 2. Fundamental Theory and Methodology

### 2.1. Blind Source Separation

BSS refers to the technique of extracting original source signals from observed mixtures in the absence of prior knowledge. More precisely, the lack of prior knowledge pertains primarily to the content of the source signals themselves and the mixing mechanisms among these signals. A classic illustration of BSS is the “cocktail party” problem, which can be delineated as follows: in a cocktail party scenario where multiple sound sources are concurrently present, the challenge consists in isolating each individual source from the composite sound mixture.

A more generalized formulation of BSS can be articulated within the framework of a multiple-input multiple-output system. In this context, “m” is considered to be the total number of independent source signals, denoted as $s_{1}\left(t\right),\;s_{2}(t),\;∆,\;s_{m}\left(t\right)$, and “n” is considered to be the total number of independent observed signals, represented as $x_{1}\left(t\right),{\;x}_{2}(t),\;∆,{\;x}_{n}\left(t\right)$. The mixing process between the source signals remains unknown. It is presumed that there exists an underlying relationship between the observed signals and the source signals, which can be mathematically expressed as follows [43]:

(3) $$x\left(t\right)=\mathrm{As}(t),$$

where A is the coefficient matrix, x(t) is the observed signal matrix, and s(t) is the source signal matrix. Given that x(t) is known, the objective is to determine an inverse system to retrieve s(t), which can be mathematically formulated as [43]:

(4) $$y\left(t\right)=\mathrm{Wx}(t),$$

where y(t) represents an estimate of the source signal s(t), and W is the recovery matrix.

In this way, the blind source separation problem is transformed, enabling the individual separation of source signals once the recovery matrix is obtained through specific solving techniques.

### 2.2. Second-Order Blind Separation

SOBI [34] is a BSS algorithm based on second-order statistics. It is an evolution of the AMUSE (Algorithm for Multiple Unknown Signals Extraction) [44], utilizing the eigenvalues and eigenvectors of the zero-delay covariance matrix to whiten the signal. Subsequently, it employs the eigenvectors of the delayed covariance matrix of the whitened signal to estimate the source signals. In constructing the covariance matrix, SOBI uses a multi-step delay, unlike the single-step delay of the AMUSE. This modification decreases sensitivity to delay selection and enhances the algorithm’s stability. Furthermore, SOBI incorporates innovations from the JADE (Joint Approximate Diagonalization of Eigen matrices) [43] algorithm in computing the recovery matrix, and it applies this joint approximate diagonalization method to the covariance matrix to derive a unitary matrix for recovering the mixing matrix. These optimizations significantly minimize errors related to delay selection while retaining the advantages of the AMUSE. Consequently, SOBI improves the separation performance of the algorithm and reduces sensitivity to outliers, regardless of whether the source signals follow a Gaussian distribution.

The steps of the SOBI are outlined below [39]:

1. Based on Equation (3), perform whitening on x(t):
  (5) $$\tilde{x}\left(t\right)=\mathrm{Vx}(t),$$
  where V is the whitening matrix that minimizes the correlation between the components of the $\tilde{x}\left(t\right)$ signal and transforms its covariance matrix into an identity matrix.
2. The delay covariance matrix of the source signal can be written as:
  (6) $$R_{s}\left(\tau \right)=s(t)s^{T}(t\;-\;\tau ),$$
  where $R_{s}$ represents the delay covariance matrix of s(t), and τ denotes the delay constant. The selection criteria for the integer τ involve a cyclic traversal within the range from 1 to $\tau_{\max}$, where $\tau_{\max}$ can be determined using the following equation:
  (7) $$\tau_{\max\;}=p\;\times \;{\mathrm{row}}_{x},$$
  where p represents the artificially selected initial delay parameter (typically, values of p less than 10 are generally sufficient, as excessively large values are unnecessary and can lead to decreased computational efficiency), and ${\mathrm{row}}_{x}$ indicates the number of rows in the observed signal matrix x(t). The delay covariance matrix of the source signal after whitening preprocessing can be formulated as:
  (8) $$R_{\tilde{x}}\left(\tau \right)=\tilde{\;x}\left(t\right){\tilde{x}}^{T}(t\;-\;\tau )$$
  By taking a set of distinct values $\tau_{1},\;\tau_{2},\;∆,\;\tau_{p}$ for τ, which represent different time delays, it derives:
  (9) $$R_{\tilde{x}}\left(\tau_{i}\right)=\mathrm{VAs}(t){(\mathrm{VAs}(t\;-\;\tau ))}^{T}$$
  and
  (10) $$R_{\tilde{x}}(\tau_{i})\;=UR_{s}\left(\tau_{i}\right)U^{T},$$
  where U is an orthogonal matrix, and it is known that $R_{s}(\tau_{i})$ is a diagonal matrix.
3. Based on $R_{\tilde{x}}\left(\tau \right)$, the optimal approximation algorithm is employed to find the optimal matrix U, and subsequently, the blind source separation matrix W is calculated as:
  (11) $$W=U^{T}V$$

The source signal is ultimately estimated as Equation (4).

### 2.3. Advantages of SOBI

From a mathematical perspective, SOBI operates on the principle of decorrelating the source signals, leveraging solely second-order statistics without the need for independence assumptions. Consequently, SOBI is effective in addressing the challenges associated with separating non-Gaussian signals that arise from the combination of specific harmonic sources, where other BSS algorithms, such as Independent Component Analysis (ICA), may struggle.

Furthermore, in the context of real-world physical systems, SOBI exploits the time-structured characteristics inherent in mechanical vibration signals and demonstrates resilience to both damping effects within the system and noise contamination in the mixed signals. In comparison to other signal decomposition methods, such as EMD, SOBI does not require prior knowledge to determine algorithm parameters and exhibits robust noise performance while ensuring the independence of the decomposition results. As a result, SOBI is particularly well suited for tackling complex signal design challenges in practical engineering applications.

### 2.4. TS-SOBI Method

The input structural strain, ε(t), comprises various mixed components that are mutually uncorrelated. Among these components, the temperature-induced strain $\varepsilon_{T}\left(t\right)$ and the vehicle-induced strain $\varepsilon_{V}\left(t\right)$ are the dominant factors, as illustrated in Equation (2). The primary objective of the TS-SOBI method is to separate $\varepsilon_{T}\left(t\right)$ from ε(t), thereby enabling the assessments of the variations in $\varepsilon_{V}\left(t\right)$ and other related strain components.

To guarantee the independence of the separated signals, TS-SOBI necessitates that the number of source signals collected be greater than or equal to the number of components of different properties within the source signal. In terms of the composition of strain signals, experimental results indicate that when the number of source signals reaches or exceeds four, the temperature-induced strain becomes more distinctly separable from other strain components.

Then, the whitening phase of the TS-SOBI method has been improved according to the following content based on SOBI. In practical SHM systems, sensor data often exhibit redundancy and sensors are prone to failures, which can jeopardize data collection. In such scenarios, the eigenvalues of the covariance matrix during the signal whitening process may converge towards zero, leading to numerical instability. To address this issue, TS-SOBI incorporates small regularization terms into the whitening matrix V during the whitening process outlined in Equation (5):

(12) $$\hat{V}=V+\alpha I,$$

where $\hat{V}$ represents the enhanced whitening matrix, α (a very small value, for instance, 1 × 10^−6^) is the regularization parameter, and I is the identity matrix. Subsequent to the whitening process, the input strain signal is transformed into uncorrelated strain components ${\overset{´}{\varepsilon }}_{i}\left(t\right)$, which encapsulate the characteristics of $\varepsilon_{T}\left(t\right)$ and $\varepsilon_{V}\left(t\right)$.

In the subsequent procedure, the core step of TS-SOBI, known as multi-step delay, is executed. In ε(t), the periods of $\varepsilon_{T}\left(t\right)$ are generally considered to be longer than those of $\varepsilon_{V}\left(t\right)$ [13]. This characteristic aligns well with the mechanism of multi-step delay, facilitating the separation of signals with distinct properties. In this study, the initial delay parameter in Equation (7) is set to 3. According to Equations (6)–(10), various delay constants $\tau_{i}$ are introduced to further differentiate and separate individual strain components $\varepsilon_{i}\left(t\right)$. Based on the outcomes of the multi-step delay process, the optimal approximation algorithm outlined in Equation (10) is utilized to determine the optimal strain separation matrix $W_{\varepsilon }$.

**Figure 1:**
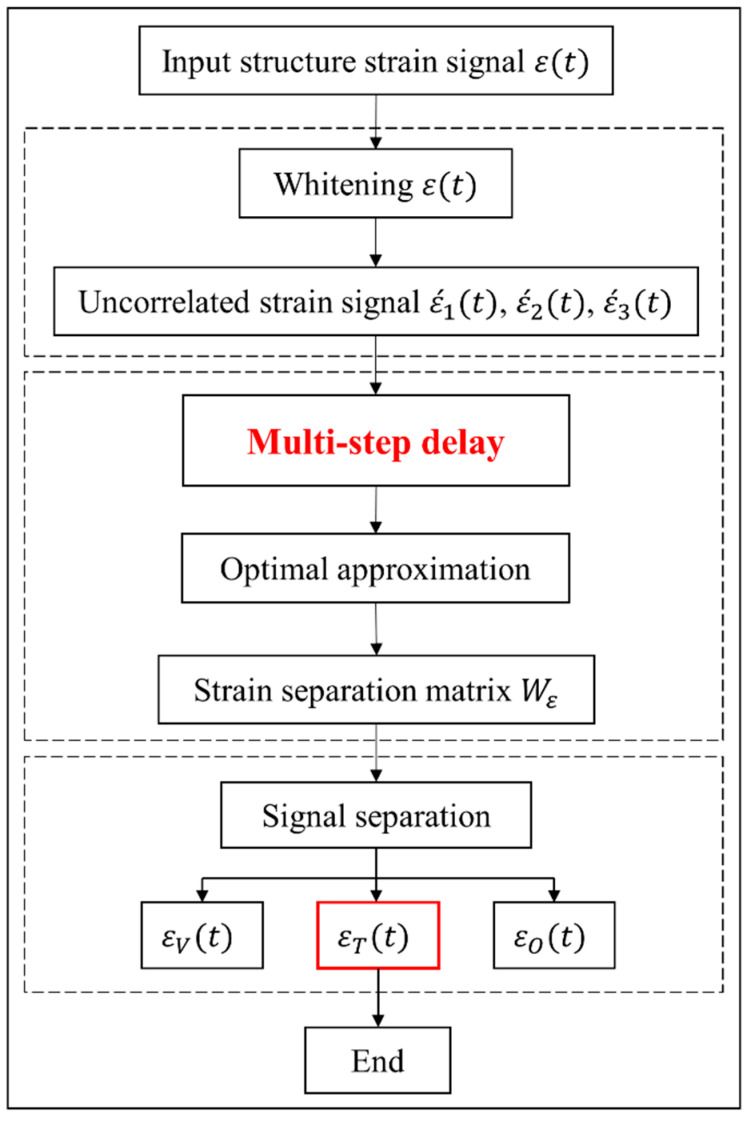
Flowchart of the TS-SOBI method.

Thus, the original strain signal ε(t) is effectively decomposed into $\varepsilon_{T}\left(t\right)$, $\varepsilon_{V}\left(t\right)$, and other components $\varepsilon_{O}\left(t\right)$, with $\varepsilon_{T}\left(t\right)$ being the primary focus of the TS-SOBI method. The complete implementation workflow of the TS-SOBI method is depicted in Figure 1, which visually outlines the three steps involved in temperature-induced signal separation and emphasizes the key processes.

## 3. Numerical Verification

### 3.1. Finite Element Model

**Figure 2:**
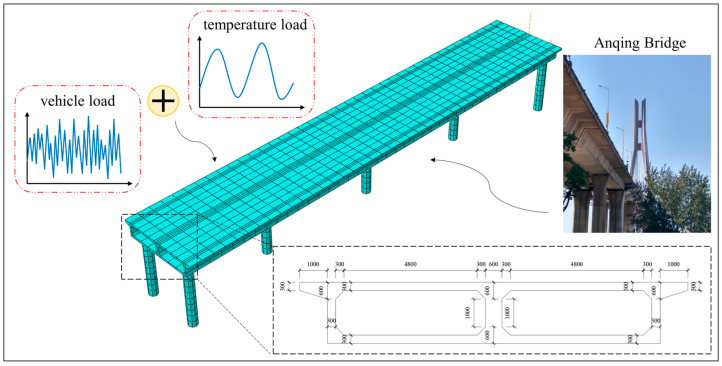
Schematic diagram and cross-section of the FE model.

**Figure 3:**
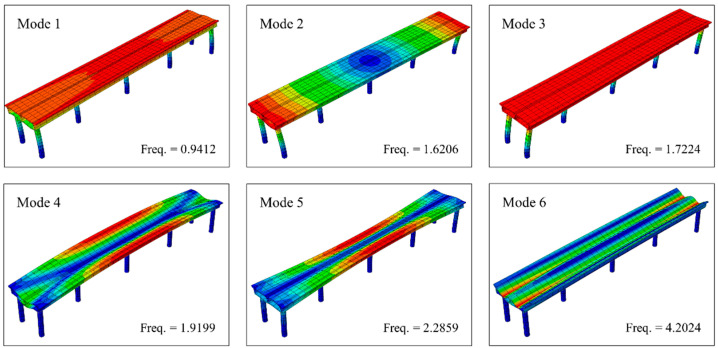
The first six modes and frequencies of the FE model. Colors represent displacement under different modes, with blue to red representing an increase in displacement values.

In the numerical verification section, an FE model of a concrete bridge is developed utilizing ABAQUS ver. 2022. This FE model is informed by the design of the approach bridge of the Anqing Yangtze River Highway Bridge, situated in Anhui Province, China. The main girder, spanning 80 m, adopts a single box girder structure with a double-chamber configuration. It rests on circular piers, which measure 10 m in height and 1.5 m in diameter. The model features double parallel piers on both sides and single-column piers aligned along the centerline at the girder’s midpoint. The boundary conditions at the pier bases are fully fixed, and the interface between the piers and the girder is modeled as a rigid connection. Both the girder and the piers are composed of Chinese grade C30 concrete, exhibiting a Poisson’s ratio of 0.2 and a density of 2400 $\mathrm{kg}/m^{3}$. The relationship between the elastic modulus and temperature employed here is derived from Jiao [45], who presented a regression analysis formula for the elastic modulus of C30 concrete within the temperature range of −20 °C to 60 °C. The FE model is meshed with 3481 nodes and 1720 elements. Figure 2 depicts a schematic diagram of the model, including its cross-section, while Figure 3 showcases the first six modal frequencies and their corresponding modal shapes.

**Figure 4:**
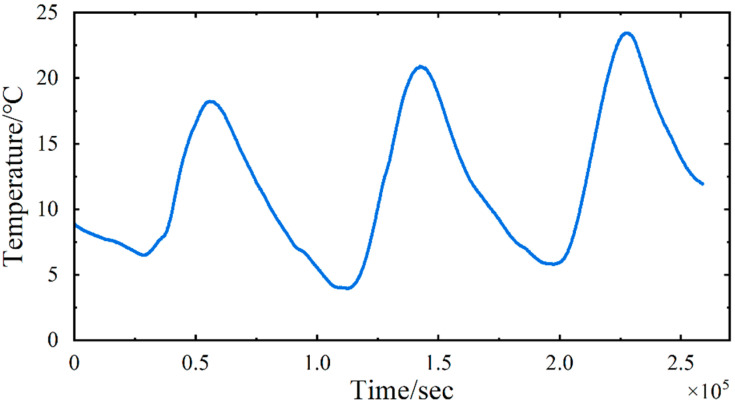
The temperature variation curve.

According to Equation (2), the FE model encompasses both temperature and vehicle loads, which are the primary factors contributing to structural strain. The temperature load is applied by adjusting the temperature change curve across the entire model and assigning a coefficient of thermal expansion of $\alpha =1\;\times \;10^{-5}$ for the concrete material. The temperature curve is set to match the actual temperature data collected from the SHM system of the Sutong Yangtze River Highway Bridge, specifically from measurements taken between 25 and 27 February (3 days) in 2018, using thermometer No. WD040120. This thermometer, which has a sampling frequency of 1 Hz, is a thermo-Q non-immersed pavement temperature sensor manufactured by Quixote Transportation Technologies in the U.S. It is installed on the central lamp post of the bridge deck, positioned in the middle of the main span. Figure 4 depicts the temperature variation curve utilized in this analysis.

Regarding vehicle loads, they manifest as moving loads of differing magnitudes in real-world traffic scenarios, generally concentrated in the center region of each lane. To streamline the loading conditions for the FE model, the model incorporates two lanes on either side of the main girder as the designated load application area.

### 3.2. Working Condition 1: Static Load

**Figure 5:**
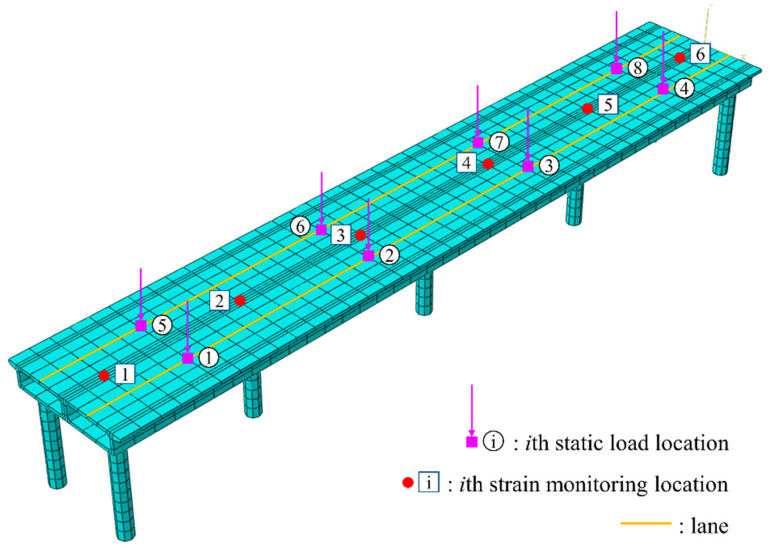
Working Condition 1: Load consideration.

**Figure 6:**
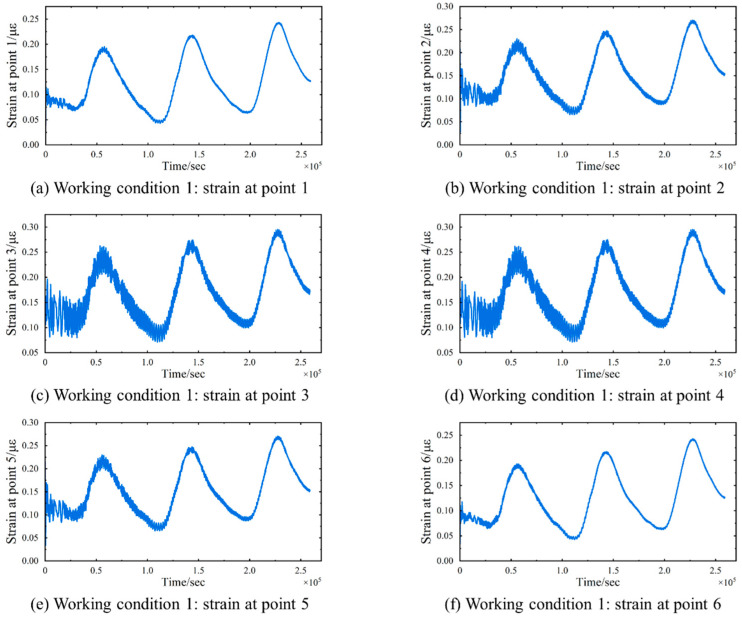
Working Condition 1: Six sets of strain signals collected. (**a**) Strain at monitoring point 1. (**b**) Strain at monitoring point 2. (**c**) Strain at monitoring point 3. (**d**) Strain at monitoring point 4. (**e**) Strain at monitoring point 5. (**f**) Strain at monitoring point 6.

In Working Condition 1, which represents a simplified load scenario, four designated points for vehicle loads are established in each lane. These points are utilized to apply various magnitudes of static concentrated loads. Figure 5 illustrates the lane configurations and the positions of the vehicle load application points, while Table 1 provides a list of the vehicle load magnitudes at each point. The FE model includes six strain data monitoring points (as depicted in Figure 5). The number of source strain signals satisfies the minimum requirements for effective temperature separation, thereby ensuring the quality of independent signal separation while mitigating the computational complexity associated with excessive data. A time history analysis is performed to obtain the strains at different collection points, recorded every five minutes over a period of three days, resulting in a total of 6 × 864 strain data signals denoted as ε(t). The six sets of strain signals obtained from the model are presented in Figure 6. All strain curves demonstrate a strong correlation with the temperature change curve, indicative of temperature loading. Furthermore, the strain curves exhibit oscillations, with the amplitude increasing as the strain data collection points approach the mid-span of the bridge.

**Figure 7:**
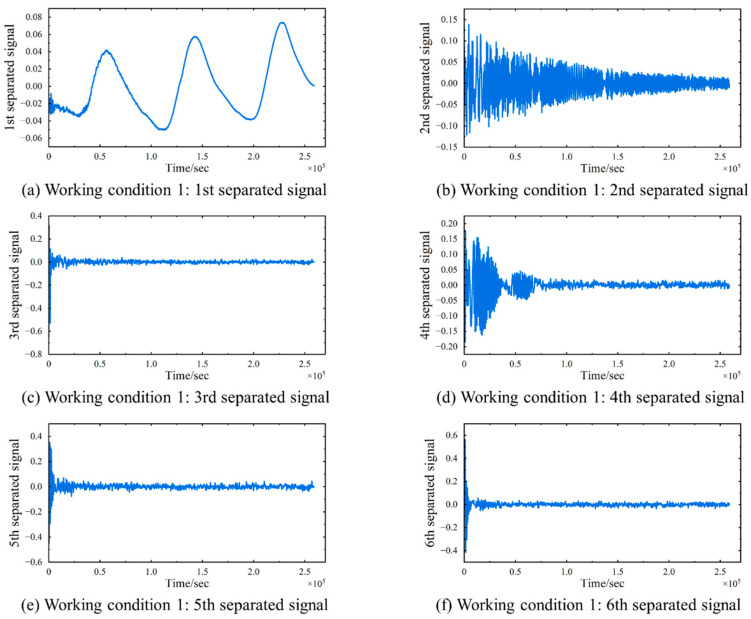
Working Condition 1: Separation results of strain signals. (**a**) The 1st separated signal. (**b**) The 2nd separated signal. (**c**) The 3rd separated signal. (**d**) The 4th separated signal. (**e**) The 5th separated signal. (**f**) The 6th separated signal.

**Figure 8:**
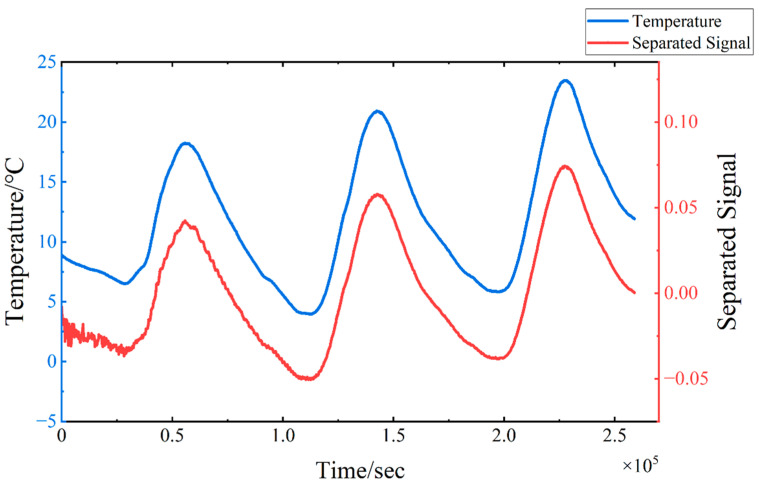
Working Condition 1: The comparison between the first separated signal and the model temperature change curve.

The TS-SOBI method is applied to isolate temperature-induced strain from the obtained six strain signals, and the separation results are depicted in Figure 7. In the figure, TS-SOBI effectively decomposes the original strain signal into six mutually independent components. The first separated signal exhibits a fluctuation pattern that closely mirrors the temperature change curve, whereas the remaining separated signals generally appear as straight lines with minimal fluctuations centered around zero. A comparison between the first separated signal and the model temperature change curve, as illustrated in Figure 8, reveals a consistent trend. Notably, the separation signal in the numerical simulation results presented in Figure 8 displays some oscillatory behavior during the initial phase. This phenomenon can be attributed to the instability of the results generated by the finite element software during the initial steps of the temperature–displacement coupling analysis. However, it is crucial to emphasize that the overall trend of the calculations aligns with the actual temperature curve. This suggests that the first separated signal predominantly reflects the strain variation induced by temperature, whereas the other separated signals may be attributable to other strain components. These findings demonstrate that TS-SOBI successfully segregates temperature-induced strain $\varepsilon_{T}\left(t\right)$ from the original signals.

Additionally, it is essential to acknowledge that BSS methods, including the TS-SOBI method, are inherently subject to magnitude uncertainty of the independent components produced during signal decomposition. This implies that the magnitudes of the separated signals are relative, not absolute. Consequently, the *y*-axis values in Figure 7 do not represent the absolute magnitudes of $\varepsilon_{T}\left(t\right)$ or the other components. Similarly, the separation signal should be considered dimensionless, as it is derived through intricate mathematical calculations and lacks a direct correspondence to physical significance. However, this limitation does not diminish the validity of the results obtained from BSS. The primary objective of the TS-SOBI and BSS methods is to discern the characteristics of the independent component curves and analyze their temporal evolution, rather than to provide a precise quantification of their magnitudes.

### 3.3. Working Condition 2: Sudden Load

**Figure 9:**
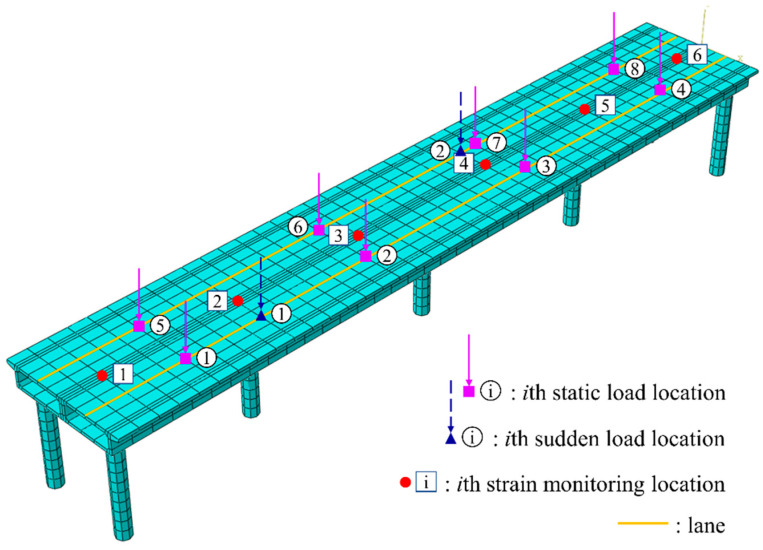
Working Condition 2: Load consideration.

**Figure 10:**
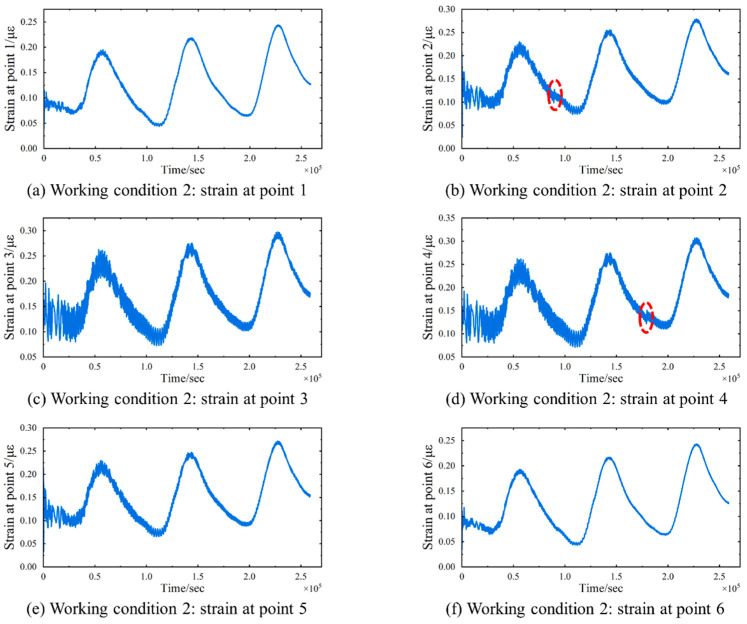
Working Condition 2: Six sets of strain signals collected. (**a**) Strain at monitoring point 1. (**b**) Strain at monitoring point 2. (**c**) Strain at monitoring point 3. (**d**) Strain at monitoring point 4. (**e**) Strain at monitoring point 5. (**f**) Strain at monitoring point 6. The red circles mark the occurrence of the sudden changes.

To further investigate the characteristics of the remaining separated signals, sudden loads are introduced in Working Condition 2, while retaining the static loads from Working Condition 1 and keeping the locations of strain monitoring points unchanged. Specifically, two sudden concentrated loads are applied adjacent to strain measurement points 2 and 4, as depicted in Figure 9. The first sudden load of 600 kN is applied at $0.9\;\times {\;10}^{5}$ s, followed by a second load of 500 kN at $1.8\;\times \;10^{5}$ s, as detailed in Table 2. Both sudden loads are substantially larger than the eight static loads and are sustained throughout the duration of the numerical simulation. A time history analysis is conducted using the same parameters as in Working Condition 1, and strain data signals ε(t) are collected from six measurement points, with the results presented in Figure 10. Analysis of the strain curves reveals that the strain at point 2 undergoes a sudden change at $0.9\;\times \;10^{5}$ s, while the strain at point 4 shows a sudden change at $1.8\;\times \;10^{5}$ s. The strain responses at other monitoring points remain largely unchanged compared with those observed in Working Condition 1. This indicates that the application of a concentrated load, despite its large numerical value, primarily influences the strain in the vicinity of the applied location, with minimal impact on strains at distant locations.

**Figure 11:**
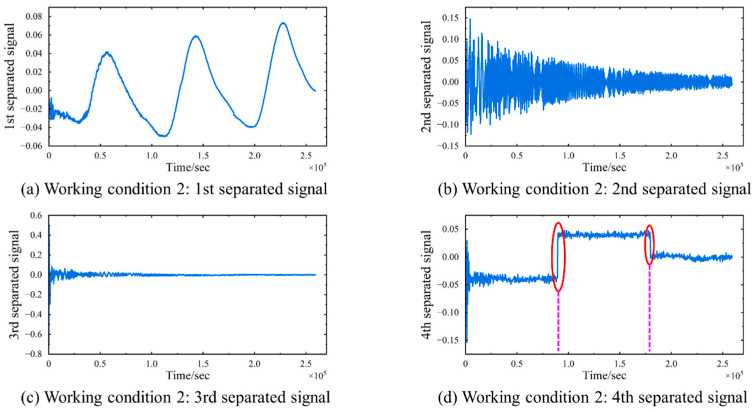
Working Condition 2: First four separated signals in separation results of strain signals. (**a**) The 1st separated signal. (**b**) The 2nd separated signal. (**c**) The 3rd separated signal. (**d**) The 4th separated signal. The red circles mark the abrupt change and the purple dashed lines indicate its occurrence time.

**Figure 12:**
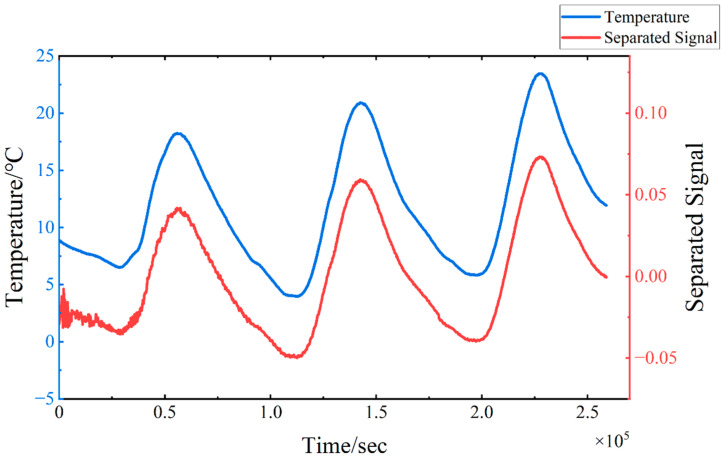
Working Condition 2: The comparison between the first separated signal and the model temperature change curve.

The strain signals undergo processing via TS-SOBI, leading to the extraction of the first four separated signals, as depicted in Figure 11. The first separated signal maintains a consistent trend with the temperature curve, and a comparison with the temperature data presented in Figure 12 confirms its temperature-induced nature. The oscillation phenomenon observed at the inception of the separated signals in Figure 12 can be attributed to the same factors as those identified in Figure 8. The second separated signal exhibits a gradual decrease in oscillation amplitude, fluctuating randomly around zero, whereas the third separated signal remains consistently at zero. Notably, the fourth signal displays prominent abrupt changes at $0.9\;\times {\;10}^{5}$ and $1.8\;\times {\;10}^{5}$ s. By correlating these observations with the timing of the load applications, a deduction can be drawn that the fourth separated signal represents the strain component induced by the external loads, with its sudden changes indicating the instants of sudden load application. The third separated signal can be interpreted as the strain component associated with the deadweight of the model, which remains constant over time. Furthermore, the second separated signal can be identified as representing minor fluctuation errors stemming from the simulation software.

The strain separation results for Working Condition 2 underscore the effectiveness of the TS-SOBI method in isolating the temperature-induced component from the overall strain. Furthermore, TS-SOBI facilitates the identification of changes in load application by examining the variations in the remaining strain components. These variations can be utilized in assessing the vehicle-induced strain $\varepsilon_{V}\left(t\right)$, providing insights into load considerations.

## 4. Experimental Validation

### 4.1. Structural Strain Collection

The TS-SOBI method is further validated through its application to actual SHM data from the Sutong Yangtze River Highway Bridge, located in Jiangsu Province, China. The primary monitored indices of the bridge’s SHM system encompass dynamic strains, temperatures, accelerations, wind characteristics, corrosion, and other relevant parameters.

**Figure 13:**
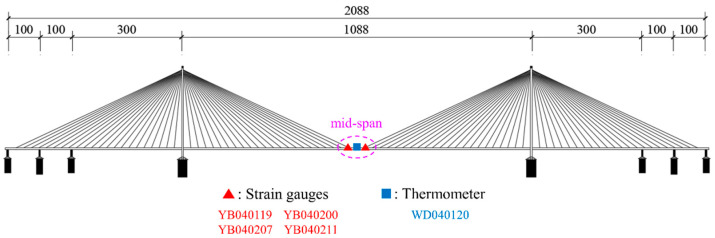
Experimental validation: Location map of sensors using data.

**Figure 14:**
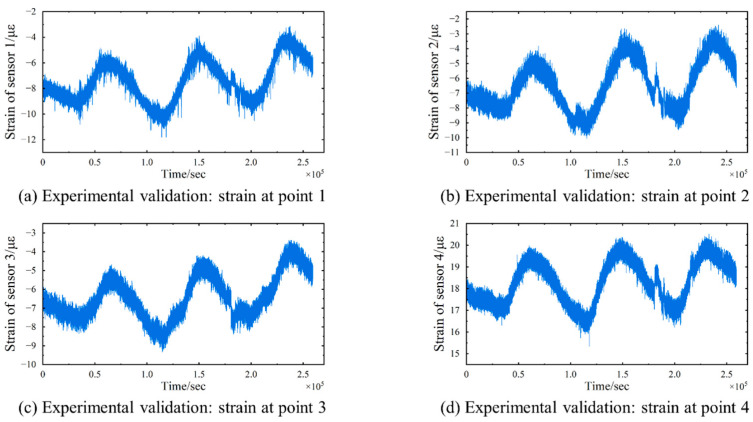
Experimental validation: Four sets of strain signals collected from the strain gauges. (**a**) Strain of strain gauge 1. (**b**) Strain of strain gauge 2. (**c**) Strain of strain gauge 3. (**d**) Strain of strain gauge 4.

**Figure 15:**
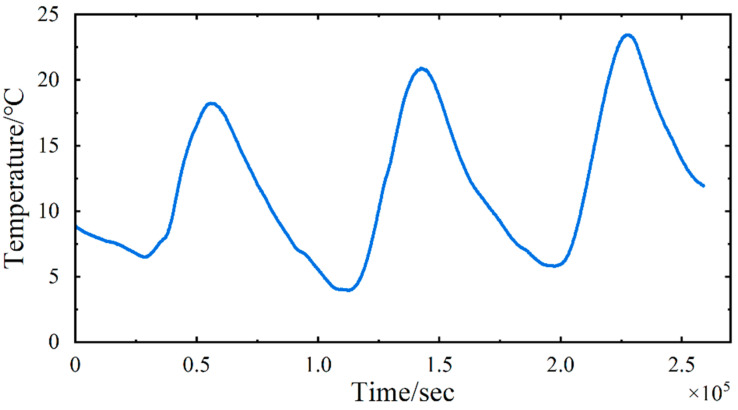
Experimental validation: The temperature variation curve of the bridge.

This study utilized data from five sensors: four strain gauges (Nos. YB040119, YB040200, YB040207, YB040211) and one thermometer (No. WD040120). The strain gauges are positioned on the downstream side of each steel truss, distributed on the mid-span section of the bridge, whereas the thermometer is mounted on the central lamp post of the bridge deck, precisely situated at the midpoint of the main span, with their respective locations depicted in Figure 13. The selection of these strain gauges was necessitated by the paramount significance of the mid-span location in cable-stayed bridges, rendering their monitoring indispensable. Furthermore, the data obtained from these four strain gauges demonstrate a comparatively lower incidence of errors. The data were collected over a 72 h period, from 25 to 27 February, 2018, with a sampling rate of twenty measurements per second (20 Hz) for the strain gauges and one measurement per second (1 Hz, as mentioned above) for the thermometer. The obtained strain data are presented in Figure 14, while the temperature curve is illustrated in Figure 15.

### 4.2. Structural Strain Separation with TS-SOBI

**Figure 16:**
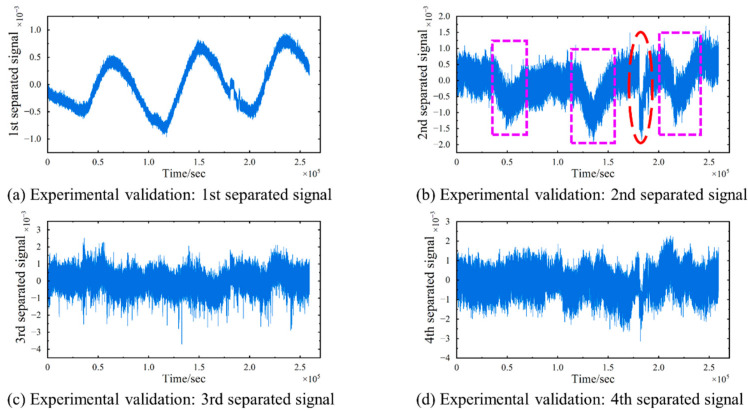
Experimental validation: Separation results of strain signals. (**a**) The 1st separated signal. (**b**) The 2nd separated signal. (**c**) The 3rd separated signal. (**d**) The 4th separated signal. The purple rectangles mark the existence of downward depressions, and the red circle marks the occurrence of sudden change.

**Figure 17:**
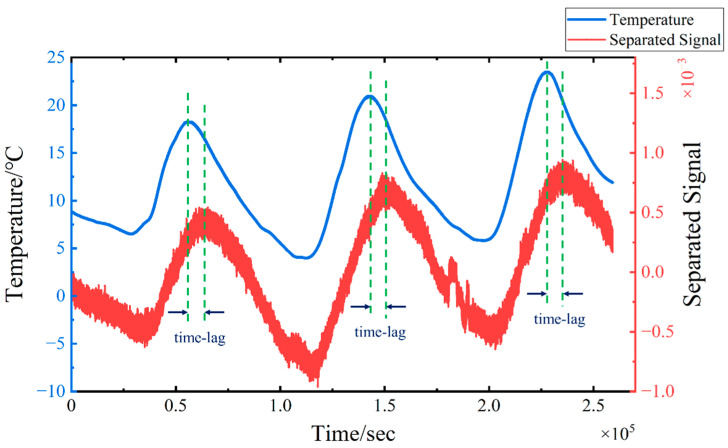
Experimental validation: The comparison between the first separated signal and the model temperature change curve.

The TS-SOBI method is applied to the four measured strain signals to separate the temperature-induced strain. The results of the four separated signals are presented in Figure 16. On the one hand, the first separated signal closely resembles the pattern of the temperature change curve, which can be interpreted as the temperature-induced strain curve $\varepsilon_{T}\left(t\right)$. Furthermore, a comparison between the variation of temperature-induced strain and temperature is illustrated in Figure 17. Both exhibit similar trends; however, it is crucial to note that a significant time-lag effect is observed in the waveforms of the two curves. This delay is attributed to the inherent lag in the structural response to temperature variations in real-world engineering environments [46], which must be taken into account when assessing the impact of temperature-induced strains.

**Figure 18:**
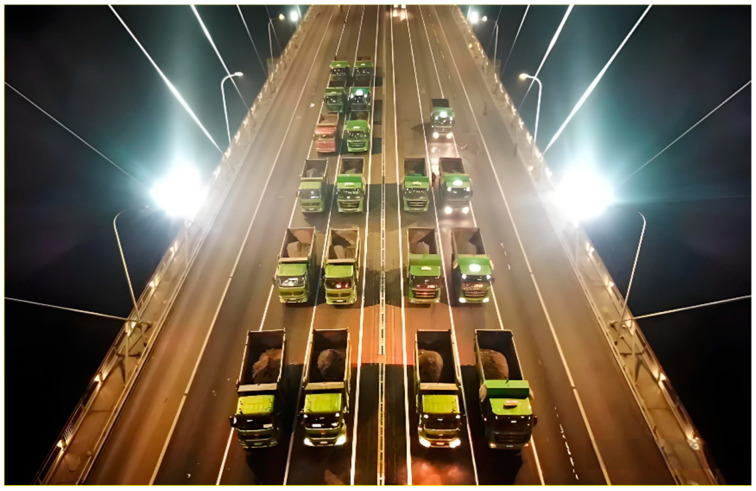
Experimental validation: Experimental load-bearing trucks full of sand and stones on Sutong Bridge.

**Figure 19:**
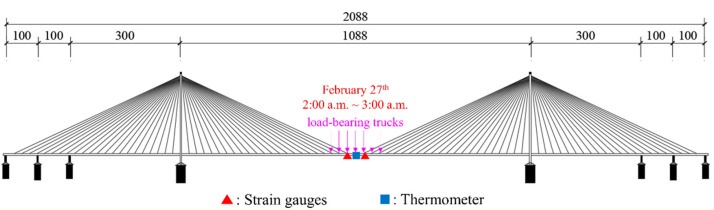
Experimental validation: Locations of load-bearing trucks in the static load test.

On the other hand, the second separation signal exhibits three distinct downward depressions (highlighted by purple rectangles), occurring at approximately 13 h, 36 h (12:00 p.m. on the second day), and 61 h (1:00 p.m. on the third day). These depressions are characterized by an initial downward fluctuation followed by an upward fluctuation in the surrounding area. The signal characteristics correspond well with the actual traffic flow on the bridge: specifically, traffic flow gradually increases in the morning, peaks at noon, and then decreases. Additionally, a brief yet prominent sudden change (highlighted by a red circle) is observed around the 50th hour (2:00 a.m. on the third day). This aligns with the fact that a static load test was conducted on the Sutong Bridge at 2:00 a.m. on February 27th, during which 52 load-bearing trucks (as shown in Figure 18) accessed the bridge in quick succession and remained in the mid-span region of the bridge for approximately one hour (as shown in Figure 19). This correlation strongly supports the conclusion that the second separation signal reflects vehicle-induced strain $\varepsilon_{V}\left(t\right)$, thereby enabling the determination of variations in vehicle load on the bridge. As for the subsequent separated signals, they generally exhibit irregular fluctuations around zero, which can be attributed to strains induced by other contributing factors $\varepsilon_{O}\left(t\right)$, such as shrinkage and dead loads.

Thus, the validation of TS-SOBI using actual bridge sensor data has been successfully accomplished. TS-SOBI not only effectively separates temperature-induced strain but also precisely captures the variations in bridge load as reflected in the separated vehicle-induced strain. This method offers substantial value for engineering applications.

## 5. Conclusions

To effectively address the temperature effects in strain data collected from the bridge SHM system, this study investigates a novel separation method based on an improved BSS technique, termed TS-SOBI. Through validations using numerical simulation and application of real bridge strain data, the following conclusions can be drawn:

1. TS-SOBI optimizes the determination of the whitening matrix within the framework of SOBI by utilizing a multi-step time delay mechanism that is better suited to the problem of strain separation.
2. In the numerical simulation, TS-SOBI effectively accomplishes the separation of temperature-induced strain under both static and sudden loading conditions. Furthermore, in the validation using real bridge data, the separation results obtained with TS-SOBI demonstrate a consistent time-lag phenomenon when compared with the actual bridge temperature curve, further confirming the validity of this method.
3. After separating the temperature-induced strains using the TS-SOBI method, the remaining strain components can indicate changes in load conditions. This aligns with the sudden load changes observed in the numerical simulations and the daily traffic load trends identified during the validation with real bridge data. Consequently, TS-SOBI exhibits significant potential for offering critical insights into the overall health status of the bridge.

In future research, the separation results obtained through TS-SOBI can be further refined and analyzed by leveraging intrinsic mathematical properties of the strain components. The objective is to improve the representation of temperature-induced strains, vehicle-induced strains, and other components in more complex loading scenarios.

## Figures and Tables

**Table 1:** Working Condition 1: Magnitudes of loads at different points.

Application Point Number & Type	Magnitude of Vehicle Load/kN
1-static	16.236
2-static	61.123
3-static	23.671
4-static	36.857
5-static	31.405
6-static	32.500
7-static	78.913
8-static	20.570

**Table 2:** Working Condition 2: Magnitudes of static and sudden loads at different points.

Application Point Number & Type	Magnitude of Vehicle Load/kN
1-static	16.236
2-static	61.123
3-static	23.671
4-static	36.857
5-static	31.405
6-static	32.500
7-static	78.913
8-static	20.570
$1-\mathrm{sudden}\;(\mathrm{active}\;\mathrm{at}\;0.9\;\times {\;10}^{5}$ s)	600.0
$2-\mathrm{sudden}\;(\mathrm{active}\;\mathrm{at}\;1.8\;\times \;10^{5}$ s)	500.0

## Data Availability

Data are unavailable due to privacy restrictions.
